# Physiochemical characteristics and sensory properties of plant protein isolates–konjac glucomannan compound gels

**DOI:** 10.1002/fsn3.3471

**Published:** 2023-06-09

**Authors:** Yueying Yao, Wenmeng He, Baojun Xu

**Affiliations:** ^1^ Food Science and Technology Program, Department of Life Sciences BNU‐HKBU United International College Zhuhai China; ^2^ Guangdong Provincial Key Laboratory of Interdisciplinary Research and Application for Data Science BNU‐HKBU United International College Zhuhai China

**Keywords:** compound gels, konjac glucomannan, pea protein isolate, peanut protein isolate, physiochemical properties, soy protein isolate

## Abstract

In this study, the effects of konjac glucomannan (KGM) at different concentrations on the physiochemical and sensory properties of soy protein isolate (SPI), pea protein isolate (PPI), or peanut protein isolate (PNPI) compound gels were investigated. The results revealed that when the ratio of PNPI to KGM was 90:10, the denaturation temperature of PNPI could be significantly enhanced to 119.32°C by KGM modification. Concerning the textural and microstructural features, the amount of KGM addition had positive correlation with the hardness and chewiness of each compound gel, however, too much KGM addition will cause the unstable internal structure of the PNPI/KGM compound gels (70:30 and 60:40). Furthermore, sensory results indicated that PNPI/KGM (80:20), PPI/KGM (80:20), SPI/KGM (80:20) had great potential to be considered as prototypes for novel plant‐based products, which generated the highest acceptance scores of 5.04, 5.94, and 5.36 in each group, respectively.

## Graphical abstract

Physicochemical and sensory properties of konjac glucomannan (KGM) and protein isolate compound gels were studied. Adding optimal amount of KGM enhanced the thermal, textural, morphological, and sensory properties of gels. Compound gels received the highest consumer acceptance when protein to KGM was 80:20.

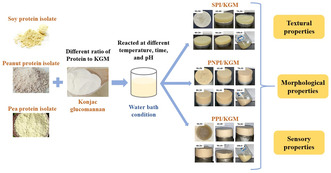

## 1 INTRODUCTION

In recent years, consumer demand for food has begun to shift from animal‐based food to plant‐based food as social trends are emphasizing health and wellness (Leyland, 2018). Many food companies seized the opportunity to innovate novel products, such as Love Raw promoted vegan chocolate, Beyond Meat developed and improved meat analog, and Food Nation introduced plant‐based patty. These food products are composed of diverse ingredients, but proteins and polysaccharides usually play vital parts in improving the texture, structure, and stability of products. For instance, proteins contribute to the formation of emulsions, foams, and gels (He et al., 2014; Mesa et al., 2009) while polysaccharides have remarkable water‐holding capacity, thickening property, and biodegradability, which have been regularly employed as gelling agents, stabilizers, thickeners, and texture modifiers (Jiang et al., 2019; Saha & Bhattacharya, 2010; Zhao et al., 2018). Several types of plant proteins and polysaccharides have been applied in the food industry, like soy protein isolate (SPI) and konjac glucomannan (KGM) are widely used as nonmeat ingredients of various meat analog products (Gao et al., 2015; Jiménez‐Colmenero et al., 2012). However, other proteins such as peanut protein isolate (PNPI) and pea protein isolate (PPI) only occupy a relatively small part of the market due to their functional characters are incompletely discovered and developed.

Isolated soy protein is considered as the most functional of the soy protein due to its desirable physiochemical and functional properties like providing elastic gel texture, emulsifying lipid and water, and controlling viscosity (Renkema et al., 2002; Singh et al., 2008). Thus, it is a favorable ingredient for lots of food‐related products, such as meat analogs and extenders, protein‐rich beverages, and bio‐based packaging (Hassan et al., 2010; Slaon, 2000). Peanut protein isolate, which contains at least 50% high‐quality protein, not only has an attractive aroma but also comprises a variety of essential amino acids that can be easily digested and absorbed by the human body. Another preferred plant protein ingredient refers to pea protein isolate, which contains more than 80% protein (Shand et al., 2007). And based on the essential amino acid composition of pea protein, its consumption can effectively assist in replenishing lysine, an essential nutrient that is deficient in cereal grains (Adebiyi & Aluko, 2011; Boye et al., 2010). High nutritional value, non‐allergenic characteristics, and great functional properties make pea protein isolate as a potential popular new additive in the food industry (Kowalczyk & Baraniak, 2011; Lam et al., 2018). The application of peanut and pea protein in food products, however, is limited due to its weak functionality as a food ingredient (Sun & Arntfield, 2010). To expand the application of these plant proteins in the food field, several chemical or physical modifications have been employed, such as associating these plant proteins with other macromolecules, including KGM (Widjanarko et al., 2011). In this way, peanut and pea protein can play an important role as substitutes for meat protein or as nutritious and functional additives, similar to what is done with soy protein (He et al., 2014; Sun & Arntfield, 2010).

Konjac glucomannan is a natural, non‐ionic, water‐soluble polysaccharide polymerized by the β‐1, 4‐linked d‐glucose and d‐mannose in the ratio of 1:1.6 (Zhang et al., 2001), and is extracted from konjac flour of *Amorphophallus konjac* C. Koch. It is a hydrocolloid dietary fiber applied as traditional food, medicinal material, and packaging component not only due to its health benefits but also its functional properties such as film‐forming, water absorptivity, thickening, stability, and emulsifying properties (Devaraj et al., 2019). Besides, the unique molecular structure of KGM contributes to its wide applications, since the presence of C–O groups in KGM provides the opportunities for combining it with other polymers, such as protein, which contains –NH_2_, –OH, –COOH, or –CONH_2_, via hydrogen bonding (Xu et al., 2007). For this reason, the value of studying the interaction between proteins and polysaccharides has become noticeable since the recombinant system may exhibit some peculiar properties.

Previous studies have revealed that many functional properties of proteins could be changed or even improved by the addition of polysaccharides which will eventually affect the texture and rheological characteristics of final products. Ding et al. (2003) proved that the addition of KGM to the SPI dispersion system, not only showed obvious synergistic thickening effect and great gelatinization ability, but also enhanced emulsifying properties of SPI. Several scholars also demonstrated that KGM could efficiently improve the stability of the soy protein emulsion (Wang et al., 2016). Besides, many researchers have explained that special intermolecular structures formed by proteins and polysaccharides not only can mimic fats but also improve the functional characteristics of food products, providing scientific support for developing low‐fat or reduced‐fat foods (Gao et al., 2015; Zhao et al., 2020). For instance, Gao et al. (2015) indicated that the combination of soy protein isolate and κ‐carrageenan can effectively improve functional properties in chopped low‐fat pork batters without weakening product quality as a more continuous structure formed. In fact, most researchers have conducted the determination of physicochemical properties of SPI/KGM mixtures due to the satisfactory functional properties and high nutritional value of these two components (Lin & Mei, 2000), while only a few studies have looked for other protein sources such as PNPI and PPI. But other proteins combined with polysaccharides also can exhibit remarkable functionality. Liu et al. (2010) found that the complex of PPI and gum Arabic exhibited a better emulsion and foam stability than the single PPI dispersion system, and the minimum pH‐solubility profile of PPI was broadened to more acidic pH values by adding of the gum Arabic. Wei et al. (2020) reported that corn fiber gum, high‐methoxy pectin, carboxymethyl cellulose, and konjac glucomannan can effectively improve the stability of pea protein dispersions to increase the utilization of pea protein in the food industry. Also, the addition of polyanionic polysaccharides could improve the stability of protein acid beverage because they can form electrostatic complex with positively charged proteins such as pea protein isolate which was previously reported by Wei and Zhang (2017). Thus, the knowledge of physicochemical aspects of the interaction of proteins and polysaccharides can further evaluate the potential application of protein‐polysaccharide mixtures in the food industry.

Therefore, the aim of this project was to systematically analyze both physiochemical and sensory properties of raw materials or final products of the SPI/KGM, PNPI/KGM, and PPI/KGM, so as to provide the scientific basis for the future research and development of the plant‐based food products.

## 2 MATERIALS AND METHODS

### 2.1 Materials

The commercial products of PNPI (protein content ≥80%), PPI (protein content ≥80%), SPI (protein content ≥90%), and KGM were purchased from Wuhan Penglei Biotechnology Co., Ltd., and used without further treatment. All chemical reagents that used in detecting physiochemical characteristics of samples were analytical grade (Sigma‐Aldrich Co.). Subsequently, all raw materials and equipment used for conducting sensory evaluation were food grade.

### 2.2 Determination of thermal properties

Thermal properties of samples were examined using a previously reported method with an adaptation (Jiang et al., 2019). After the protein and KGM powders (6 mg) were mixed in the aluminum pot of a differential scanning calorimeter (TA‐Q20 DSC; Mettler‐Toledo), 14 μL deionized water (DI water) was added with a micro syringe. Then, the mixture should be incubated at 4°C for 2 h under the sealed condition to ensure equilibration of sample and water. Whereafter, the samples were scanned from 35 to 150°C (for the single protein) or 50 to 180°C (for the mixture) at a heating rate of 5°C/min, and a sealed empty pot should be used as a reference. The thermodynamic transition temperatures namely onset (*T*_o_), peak denaturation (*T*_d_), and ending (*T*_e_) temperature were computed automatically from the thermograms using software.

### 2.3 Rapid viscosity analysis

The viscosity of each type of protein and polysaccharide conjugate was evaluated by employing Rapid Visco‐Analyzer (4500, Warriewood, Australia) based on a method of Wang et al. (2018) with slightly modification. Before analysis, each sample (4.5 g) was added by 24 mL of DI water and was detected under the programmed heating and cooling cycle. The mixed slurries were kept at 50°C for 1 min, heated to 80°C at a rate of 8.6°C/min and held at 80°C for 5 min before cooling to 30°C at 9.1°C/min and allowed to stay at 30°C for 5 min. In this study, peak temperature 1 (*PT1*), peak viscosity 1 (*PV1*), peak temperature 2 (*PT2*), peak viscosity 2 (*PV2*), trough viscosity (*TV*), trough temperature (*TT*), and final viscosity (*FV*) of the various reconstituted proteins were studied.

### 2.4 Preparation of compound gels for texture and microstructural analysis

The concentration of protein and polysaccharide conjugates was 10 g/100 mL. PNPI/KGM at different ratios, 100:0, 90:10, 80:20, 70:30, 60:40, and 50:50 was included, denoted as PNPI, PNK1, PNK2, PNK3, PNK4, and PNK5, respectively. The preparation of PPI/KGM and SPI/KGM followed the same rules, and the samples were labeled as PPI, PK1, PK2, PK3, PK4, PK5, SPI, SK1, SK2, SK3, SK4, and SK5, respectively. In short, solutions were prepared by thoroughly dissolving PNPI, or PPI, or SPI powder and KGM powder in 100 mL DI water. Besides, the homogeneous solution should be prepared by constant stirring with a magnetic stirring apparatus at room temperature for 45 min. Since pH plays an important part in various factors affecting the electrostatic interactions between proteins and polysaccharides, the pH of each kind of mixed solution was modified to its optimal pH (pH 9.0 ± 0.1 for PNPI, pH 7.1 ± 0.1 for PPI, pH 9.0 ± 0.1 for SPI) by using 2 mol/L HCl and 10% NaOH based on previous work (Feng & Wu, 2009; Gong et al., 2005; Shand et al., 2007). Then, the dispersion was stored overnight in a refrigerator at 4°C to ensure an even hydration of the protein (Cornet et al., 2020).

### 2.5 Texture profile analysis

Textural attributes of compound gels were determined applying the method of Jiang et al. (2019). After removing from the refrigerator and then cooling to the room temperature, the sample solution was transported in aluminum casings (50 mm in diameter). Sample were heated via using a water bath at temperature of 85°C for 40 min for PNPI/KGM (Feng & Wu, 2009), 93°C for 45 min for PPI/KGM (Shand et al., 2007), and 90°C for 40 min for SPI/KGM (Gong et al., 2005). Whereafter, the samples should be quickly cooled down to room temperature and followed by 20 min sonification for eliminating air bubble. After 24 h refrigeration at 4°C, the texture properties of compound gels could be analyzed until the temperature of gels equaled to 25°C for 60 min. Subsequently, a texture analyzer (Brookfield CT3; AMETEK Commercial Enterprise Co. Ltd.) was applied to measure cylindrical gel samples (20 mm in height and 50 mm in diameter) employing a TA 11/1000 probe. The operating mode was texture profile analysis (TPA), the trigger force was 5.0 g, the test speed was 0.5 mm/s, and the deformation distance was 7.5 mm. The TPA parameters namely hardness, cohesiveness, springiness, and chewiness, were computed and recorded automatically by the instrument.

### 2.6 Microstructure analysis

The microstructural features of the compound gels were measured by scanning electron microscope (SEM, ZEISS Sigma 500; ZEISS) following a procedure at Yuan et al. (2018) with adaptations. Freshly prepared compound gels were lyophilized by a vacuum freeze dryer at –50°C before observation. Then, the freeze‐dried compound gel was placed on a microscope stub and coated with platinum. Finally, the observation was performed by applying SEM with a potential accelerator of 5 kV and magnified 500× times.

### 2.7 Sensory evaluation

#### 2.7.1 Food‐grade sample preparation for sensory evaluation

The protein and polysaccharide conjugates were prepared at a constant concentration of 10 g/100 mL. PNPI/KGM at different ratios, including 90:10, 80:20, 70:30, and 60:40 denoted as PNK1, PNK2, PNK3, and PNK4, respectively. The preparation of PPI/KGM and SPI/KGM followed the same rules, and the samples were labelled as PK1, PK2, PK3, PK4, SK1, SK2, SK3, and SK4, respectively. In short, solutions were prepared by thoroughly dissolving PNPI, or PPI, or SPI powder and KGM powder in 1000 mL drinking water. Besides, a homogeneous solution was prepared by constant stirring with a blender at room temperature for 45 min. The pH of each kind of mixed solution was modified to its optimal pH (pH 9.0 ± 0.1 for PNPI, pH 7.1 ± 0.1 for PPI, pH 9.0 ± 0.1 for SPI) by using water with different pH. Sample were heated via using a water bath at temperature of 85°C for 40 min for PNPI/KGM (Feng & Wu, 2009), 93°C for 45 min for PPI/KGM (Shand et al., 2007), and 90°C for 40 min for SPI/KGM (Gong et al., 2005). Finally, the samples should be rapidly cooled to room temperature and refrigerated at 4°C until use. Moreover, the temperature of samples should equal to room temperature for 60 min before the sensory evaluation.

#### 2.7.2 Flash profile test

Fifty participants (25 females and 25 males; aged from 18 to 39 years) were selected based on their healthy status and frequency of consumption of plant protein products. The intensive of color, aroma, texture, and odor flavor of each type of gel sample with different ratio of protein to polysaccharide were assessed. A whole set of twelve different formulated samples (PNK3, PK1, PK4, SK4, SK2, PK3, PNK4, PNK1, SK1, SK3, PNK2, PK2 labeled with letters A, B, C, D, E, F, G, H, I, J, K, L, respectively) were presented simultaneously for each participant. They were required to rank the intensity of these samples for each attribute via applying an unstructured and anchored scale of 9 cm based on the “low” and “high” extremes (Albert et al., 2011; Marques et al., 2019). The ranking data were collected using the questionnaire shown in Appendix 1.

#### 2.7.3 Consumer acceptance test

The quality characteristics of gel samples were evaluated by conducting the consumer acceptance test according to the methodology described by Wangcharoen et al. (2016) with rational adaptation. The twelve samples PNK3, PK1, PK4, SK4, SK2, PK3, PNK4, PNK1, SK1, SK3, PNK2, PK2 labeled with three random digital code number were presented and evaluated by the same 50 participants selected in flash profile test using a 9‐point hedonic scale (1 = dislike extremely, 5 = neither like nor dislike, 9 = like extremely). Consumers’ acceptance for appearance, aroma, texture, taste/flavor, and overall liking of the samples were collected using the questionnaire shown in the Appendix 2.

### 2.8 Statistical analysis

All data reported in the analysis section on the physiochemical properties of samples were triplicate observations and were represented as a mean value ± standard deviation with one‐way analysis of variance. Duncan's Multiple Range Test was performed to measure the significant differences among the mean values (*p* < .05). The statistical analysis was performed using SPSS 21 software, version 21.0 (SPSS; IBM). Additionally, generalized procrustes analysis (GPA) was employed for reducing the scale effects and obtaining a consensus configuration between consumers’ sensory maps, which was performed via applying the XLSTAT statistical software, version 2021.3.1 (Addinsoft).

## 3 RESULTS

### 3.1 Thermal properties of different protein isolates formulated with KGM

The thermal properties of different protein isolates complexed with different ratios of KGM are presented in Table 1. Among PNPI/KGM complexes, the maximums of onset temperature (*T*_o_) and denaturation temperature (*T*_d_) were observed in PNK5 (99.31°C) and PNK1 (119.32°C). Among PPI group, the highest *T*_o_ and *T*_d_ values were displayed in PPI (99.54°C) and PK3 (112.75°C). Among SPI group, the maximums of *T*_o_ and *T*_d_ were observed in SK2 (99.63°C) and SPI (107.68°C). Besides, except from PNPI/KGM complexes, the maximum values of these thermal denaturation temperatures of PPI/KGM and SPI/KGM complexes were not significantly different from those without KGM.

**TABLE 1:** Thermal properties of different konjac glucomannan‐mixed gel samples.

Samples	PI/KGM ratio (%)	Thermal properties		
		*T*_o_ (°C)	*T*_d_ (°C)	*T*_e_ (°C)
PNPI	100:0	94.90 ± 0.21^c^	112.32 ± 1.12^b^	130.66 ± 1.92^b^
PNK1	90:10	99.13 ± 0.05^a^	119.32 ± 0.01^a^	134.78 ± 2.40^a^
PNK2	80:20	98.86 ± 0.77^ab^	109.74 ± 0.71^e^	130.82 ± 1.36^b^
PNK3	70:30	98.97 ± 0.08^a^	110.75 ± 0.34^cd^	128.57 ± 0.50^c^
PNK4	60:40	98.40 ± 0.46^b^	110.25 ± 0.13^de^	124.49 ± 2.00^d^
PNK5	50:50	99.31 ± 0.08^a^	111.07 ± 0.33^c^	126.28 ± 0.40^d^
PPI	100:0	99.54 ± 0.16^a^	112.02 ± 0.06^a^	131.57 ± 5.73^a^
PK1	90:10	92.34 ± 4.70^bc^	107.73 ± 0.17^b^	124.93 ± 3.30^b^
PK2	80:20	95.82 ± 1.50^ab^	105.88 ± 1.03^d^	120.43 ± 7.18^b^
PK3	70:30	98.31 ± 0.16^a^	112.75 ± 0.86^a^	131.63 ± 0.92^a^
PK4	60:40	91.14 ± 2.62^c^	107.16 ± 1.64^bc^	123.21 ± 3.22^b^
PK5	50:50	93.48 ± 5.77^bc^	106.31 ± 1.05^cd^	124.89 ± 4.07^b^
SPI	100:0	97.21 ± 4.64^a^	107.68 ± 3.25^a^	124.24 ± 2.73^ab^
SK1	90:10	95.87 ± 2.21^a^	105.69 ± 2.07^a^	127.76 ± 4.82^a^
SK2	80:20	99.63 ± 2.63^a^	106.65 ± 0.80^a^	127.98 ± 2.51^a^
SK3	70:30	97.39 ± 0.95^a^	106.46 ± 0.62^a^	120.50 ± 0.27^b^
SK4	60:40	98.29 ± 1.04^a^	107.05 ± 0.08^a^	124.84 ± 0.56^ab^
SK5	50:50	88.07 ± 2.68^b^	107.14 ± 0.33^a^	122.19 ± 0.16^b^

*Note*: Values are expressed as mean ± SD (*n* = 3); data of different alphabets in the same column were different with statistical significance (*p* < .05).

Abbreviations: PI, protein isolate; PKn (*n* = 1, 2, 3, 4, 5), the protein contained is a pea protein isolate; PNKn (*n* = 1, 2, 3, 4, 5), the protein contained is a peanut protein isolate; PNPI, peanut protein isolate; PPI, pea protein isolate; SKn (*n* = 1, 2, 3, 4, 5), the protein contained is a soy protein isolate; SPI, soy protein isolate; *T*_d_, peak denaturation temperature; *T*_e_, ending temperature; *T*_o_, onset temperature.

### 3.2 RVA profile of different protein isolates formulated with KGM

According to Table 2, compared with all PI samples [PNPI (147.00 cP), PPI (644.00 cP), SPI (282.67 cP)], the viscosity parameters such as trough viscosity (*TV*), peak viscosity 2 (*PV2*), and final viscosity (*FV*) of complexed protein and KGM mixtures increased significantly (*p* < .05) with the addition of KGM. With the increasing percentage of KGM in the sample, *PV2* in peanut and soy groups showed a similar trend, that is, increased gradually and then decreased after reaching a peak of 1882.67 cP (PNK2) and 2375.33 cP (SK3). As for pea group, *PV2* gradually increased to 3414.67 cP, and then then fluctuated irregularly after the PK2. Then, the *TV* of PNPI group, PPI group, and SPI group ranged between 140.00 cP (PNPI) and 750.33 cP (PNK5), 229.00 cP (PPI) and 1369.33 cP (PK3), 89.33 cP (SPI), and 674.00 cP (SK3), respectively. The highest *FV* values were observed in PNK5 (1516.00 cP), PK4 (3017.33 cP), and SK3 (2169.67 cP), while the lowest values were all shown in the PI samples, such as 202.33 cP for peanut group, 419,67 cP for pea group, and 193.33 cP for soy group. Accordingly, *TV* and *FV* of peanut group gradually increased, and the *TV* and *FV* of pea or soy group displayed similar fluctuations. Moreover, the *TT* of all samples only slightly fluctuated up and down within a small range, and showed no significant difference between PNKn, PKn, or SKn (*n* = 1–5) samples and PI samples.

**TABLE 2:** RVA profiles of different PI/KGM mixed samples.

Samples	PI/KGM ratio (%)	RVA parameters						
		PV1 (cP)	PT1 (°C)	TV (cP)	TT (°C)	PV2 (cP)	PT2 (°C)	FV (cP)
PNPI	100:0	147.00 ± 43.31^e^	76.38 ± 0.68^b^	140.00 ± 42.32^e^	79.98 ± 0.03^a^	215.67 ± 72.39^d^	29.22 ± 0.08^d^	202.33 ± 66.89^e^
PNK1	90:10	175.33 ± 8.33^e^	75.98 ± 0.33^bc^	165.33 ± 7.57^e^	80.00 ± 0.00^a^	1570.67 ± 58.24^b^	39.70 ± 0.00^b^	898.00 ± 57.56^d^
PNK2	80:20	505.00 ± 13.53^d^	79.98 ± 0.03^a^	497.67 ±11.59^d^	79.93 ± 0.03^b^	1882.67 ± 6.43^a^	48.18 ± 2.10^a^	907.00 ± 14.42^d^
PNK3	70:30	589.00 ± 3.61^c^	75.75 ± 0.05^bc^	561.00 ± 5.29^c^	79.98 ± 0.03^a^	1518.00 ± 163.69^b^	47.23 ± 2.15^a^	1198.67 ± 36.09^c^
PNK4	60:40	695.33 ± 18.90^b^	75.72 ± 0.08^c^	652.33 ± 15.04^b^	80.00 ± 0.00^a^	1367.00 ± 39.05^c^	29.65 ± 0.30^d^	1327.33 ± 25.72^b^
PNK5	50:50	790.67 ± 2.08^a^	75.45 ± 0.35^c^	750.33 ± 0.58^a^	80.00 ± 0.00^a^	1551.67 ± 6.66^b^	32.70 ± 0.05^c^	1516.00 ± 2.00^a^
PPI	100:0	644.00 ± 22.72^e^	58.85 ± 0.09^d^	229.00 ± 4.58^f^	78.68 ± 1.05^b^	432.33 ± 8.08^c^	29.73 ± 0.03^d^	419.67 ± 8.02^c^
PK1	90:10	981.67 ± 16.26^d^	65.53 ± 0.28^c^	651.00 ± 38.00^e^	80.00 ± 0.00^a^	3072.00 ± 270.02^ab^	31.58 ± 0.40^c^	1435.33 ± 158.32^b^
PK2	80:20	1181.00 ± 0.00^c^	79.88 ± 0.18^a^	767.00 ± 6.24^d^	79.90 ± 0.05^a^	3414.67 ± 359.67^a^	40.15 ± 0.62^b^	1596.67 ± 214.41^b^
PK3	70:30	1416.33 ± 4.73^a^	77.20 ± 2.43^b^	1369.33 ± 31.88^a^	79.13 ± 0.78^ab^	2906.67 ± 443.09^ab^	50.48 ± 2.08^a^	1820.00 ± 431.15^b^
PK4	60:40	1350.00 ± 8.00^b^	75.75 ± 0.00^b^	1260.67 ± 9.71^b^	79.98 ± 0.03^a^	3023.67 ± 135.98^ab^	29.93 ± 0.06^d^	3017.33 ± 110.00^a^
PK5	50:50	1210.33 ± 28.73^c^	75.70 ± 0.00^b^	1126.33 ± 20.74^c^	79.98 ± 0.03^a^	2863.00 ± 106.67^b^	29.90 ± 0.05^d^	2869.00 ± 88.50^a^
SPI	100:0	282.67 ± 11.68^d^	58.83 ± 0.06^d^	89.33 ± 3.51^f^	79.65 ± 0.61^a^	193.33 ± 2.89^d^	29.42 ± 0.21^b^	193.33 ± 1.53^e^
SK1	90:10	297.00 ± 2.65^d^	71.03 ± 4.55^c^	281.67 ± 1.53^e^	79.98 ± 0.03^a^	1537.67 ± 16.80^c^	35.10 ± 0.05^b^	704.00 ± 7.55^d^
SK2	80:20	531.33 ± 20.40^c^	79.78 ± 0.12^a^	479.00 ± 12.53^d^	80.00 ± 0.00^a^	1954.00 ± 138.13^b^	47.10 ± 0.56^a^	853.00 ± 10.00^c^
SK3	70:30	694.33 ± 9.87^a^	75.65 ± 0.30^b^	674.00 ± 16.37^a^	80.00 ± 0.00^a^	2375.33 ± 95.44^a^	39.10 ± 11.66^ab^	2169.67 ± 58.23^a^
SK4	60:40	623.00 ± 8.19^b^	75.85 ± 0.48^b^	577.67 ± 8.62^c^	80.00 ± 0.00^a^	1587.33 ± 34.43^c^	39.73 ± 9.88^ab^	1480.33 ± 4.51^b^
SK5	50:50	719.00 ± 27.06^a^	75.75 ± 0.00^b^	651.33 ± 15.50^b^	79.98 ± 0.03^a^	1478.33 ± 48.50^c^	29.38 ± 0.08^b^	1455.00 ± 45.03^b^

*Note*: Values are presented as mean ± SD (*n* = 3); data of different alphabets in the same column were different with statistical significance (*p* < .05).

Abbreviations: FV, final viscosity; KGM, konjac glucomannan; PI, protein isolate; PKn (*n* = 1, 2, 3, 4, 5), the protein contained is a pea protein isolate; PNKn (*n* = 1, 2, 3, 4, 5), the protein contained is a peanut protein isolate; PNPI, peanut protein isolate; PPI, pea protein isolate; PT1, peak temperature 1; PT2, peak temperature 2; PV1, peak viscosity 1; PV2, peak viscosity 2; RVA, rapid viscosity analysis; SKn (*n* = 1, 2, 3, 4, 5), the protein contained is a soy protein isolate; SPI, soy protein isolate; TT, trough temperature; TV, trough viscosity.

### 3.3 Appearances of compound gels

The compound gels made from formulas with different ratios of protein and polysaccharides were visually observed and recorded in Figure S1. The findings indicated that the PNK1, 2, 3, 4, PK1, 2, 3, 4, and SK1, 2, 3, 4, had become smoother and firmer with the ratio of the KGM increased. Howbeit, the PI samples could not change from liquid to solid state, and the final products of the sample PNK5, PK5, and SK5 could not form a homogenous gel. In this way, the samples PNPI, PPI, SPI, PNK5, PK5, and SK5 were not able to be participated in the subsequent TPA, SEM inspections, and sensory evaluation.

### 3.4 Textural properties of compound gels

In this experiment, each sample was investigated by two‐cycle TPA method, and the data obtained were summarized in Table 3. As Table 3 shown, the hardness and chewiness of PNPI/KGM, PPI/KGM, and SPI/KGM gels showed a significant increasing trend with the increase of KGM concentrations, which corresponding to the visual observation. The PNK4, PK4, and SK4 gels had the highest hardness and chewiness, and PNK4, PK1, and SK4 had the highest cohesiveness. Also, Table 3 revealed that the springiness trend of PNPI/KGM compound gel gradually rose first and then slightly decreased and reached the peak value 7.51 ± 0.00 as the ratio of PNPI: KGM was 80:20. Besides, the springiness of PPI/KGM and SPI/KGM compound gels displayed an irregular trend and peaked as the ratio of PPI or SPI to KGM was 60:40.

**TABLE 3:** Textural properties of samples with different formulations.

Samples	PI/KGM ratio (%)	Textural parameters			
		Hardness	Cohesiveness	Springiness	Chewiness
PNPI	100:0	n.a.	n.a.	n.a.	n.a.
PNK1	90:10	367.75 ± 22.98^c^	0.24 ± 0.01^c^	6.28 ± 0.39^c^	5.38 ± 0.47^c^
PNK2	80:20	1318.00 ± 26.16^b^	0.29 ± 0.01^bc^	7.51 ± 0.00^a^	27.47 ± 0.04^b^
PNK3	70:30	2614.00 ± 263.75^a^	0.35 ± 0.04^ab^	7.22 ± 0.01^a^	65.17 ± 13.40^a^
PNK4	60:40	2772.00 ± 332.34^a^	0.41 ± 0.11^a^	6.78 ± 0.43^b^	73.94 ± 14.46^a^
PNK5	50:50	n.a.	n.a.	n.a.	n.a.
PPI	100:0	n.a.	n.a.	n.a.	n.a.
PK1	90:10	579.25 ± ± 6.72^d^	0.32 ± 0.01^a^	6.16 ± 0.54^b^	10.20 ± 0.31^d^
PK2	80:20	1786.75 ± 77.43^c^	0.23 ± 0.06^b^	7.29 ± 0.28^a^	35.68 ± 1.1^c^
PK3	70:30	2500.25 ± 443.00^b^	0.24 ± 0.03^b^	6.52 ± 0.02^b^	44.08 ± 4.32^b^
PK4	60:40	3000.25 ± 86.62^a^	0.26 ± 0.01^b^	7.49 ± 0.86^a^	55.48 ± 7.18^a^
PK5	50:50	n.a.	n.a.	n.a.	n.a.
SPI	100:0	n.a.	n.a.	n.a.	n.a.
SK1	90:10	349.50 ± 10.61^d^	0.27 ± 0.08^a^	6.83 ± 0.21^bc^	6.22 ± 1.44^d^
SK2	80:20	1325.25 ± 69.65^c^	0.21 ± 0.01^b^	6.96 ± 0.49^b^	19.13 ± 1.48^c^
SK3	70:30	2558.00 ± 1.41^b^	0.26 ± 0.01^ab^	6.53 ± 0.08^c^	40.06 ± 0.82^b^
SK4	60:40	4403.80 ± 141.85^a^	0.29 ± 0.01^a^	7.46 ± 0.18^a^	90.39 ± 1.37^a^
SK5	50:50	n.a.	n.a.	n.a.	n.a.

*Note*: Values expressed are mean ± standard deviation; data of different alphabets in the same column were different with statistical significance (*p* < .05). The final products of the sample PNPI, PPI, and SPI remained liquid, therefore, they were not involved in the TPA detection. Also, the final products of the samples PNK5, PK5, and SK5 could not form a homogenous gel and, therefore, were not involved in the TPA detection.

Abbreviations: n.a., not available; PI, protein isolate; PKn (*n* = 1, 2, 3, 4, 5), the protein contained is a pea protein isolate; PNKn (*n* = 1, 2, 3, 4, 5), the protein contained is a peanut protein isolate; PNPI, peanut protein isolate; PPI, pea protein isolate; SKn (*n* = 1, 2, 3, 4, 5), the protein contained is a soy protein isolate; SPI, soy protein isolate.

### 3.5 Microstructural characteristics of compound gels

**FIGURE 1:**
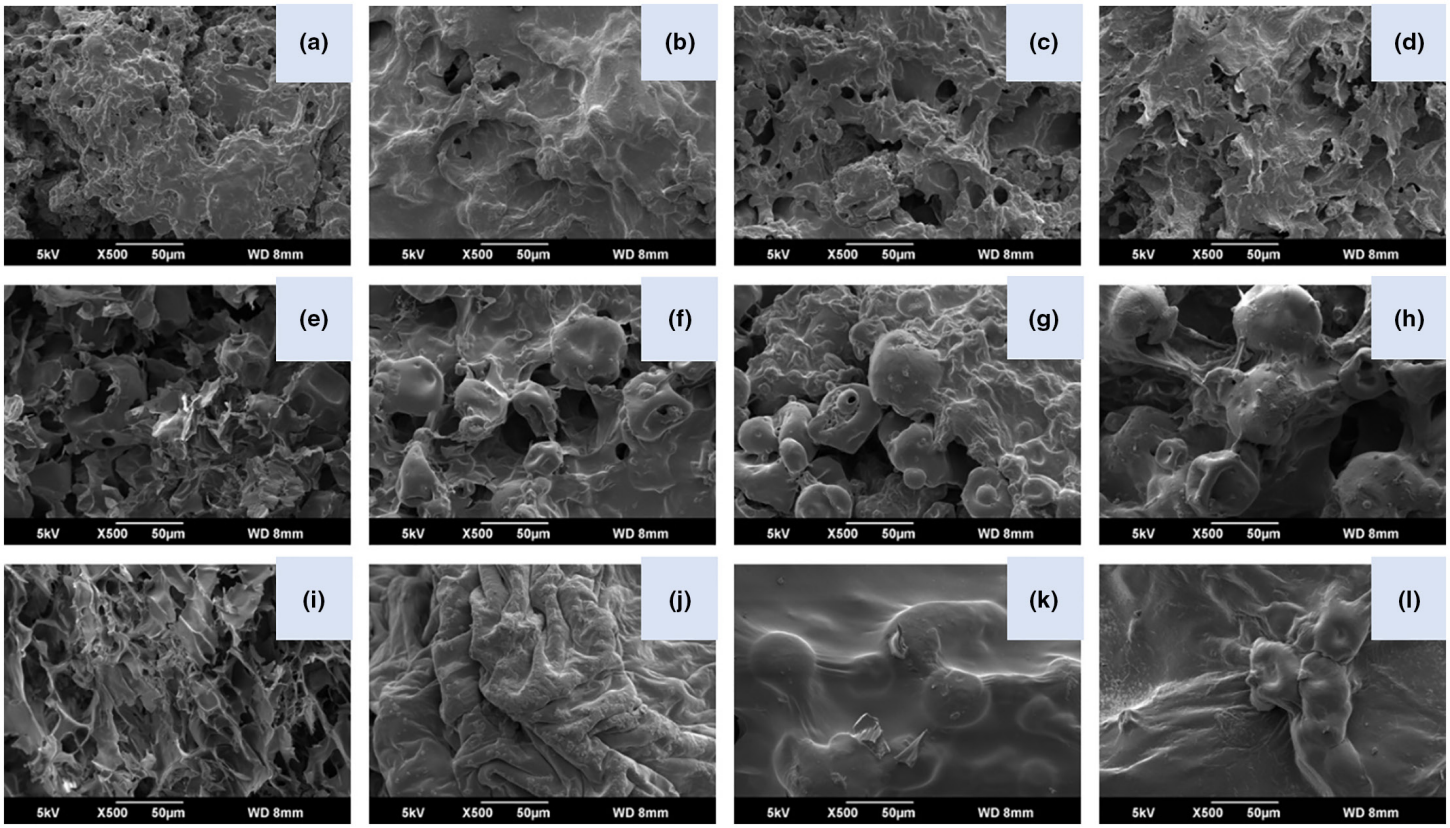
The micrographs of different gels at various protein isolate/polysaccharide ratios with a magnification of 500. Symbols including a, b, c, d, e, f, g, h, i, j, k, and l denotes PNK1, PNK2, PNK3, PNK4, PK1, PK2, PK3, PK4, SK1, SK2, SK3, and SK4, respectively.

The microstructure features of the compound gels containing different ratios of three types of protein isolates and KGM were presented in Figure 1. Among PNPI/KGM group, as the PNPI to KGM ratio was at 80:20 (Figure 1b), the gel micro‐morphology featured smoother plicated‐layer structure than PNK1 (Figure 1a). However, if the proportion of KGM continues to increase, the number of pores in the structure will increase and the structure will become rougher and more fragmented, as shown in Figure 1c,d. Besides, the PK1 (Figure 1e), and SK1 (Figure 1i) gels were fluffy, coarse, and had irregular sizes of pores in the structure after freeze‐drying. But as the content of KGM increased, less pores in the structure, the smoothness of the surface increased, and the polymer size became larger as well (Figure 1e–l). Among twelve samples, SK3 (Figure 1k) and SK4 (Figure 1l) showed the smoothest texture.

### 3.6 Sensory evaluation of compound gels

#### 3.6.1 Flash profile of compound gels

**FIGURE 2:**
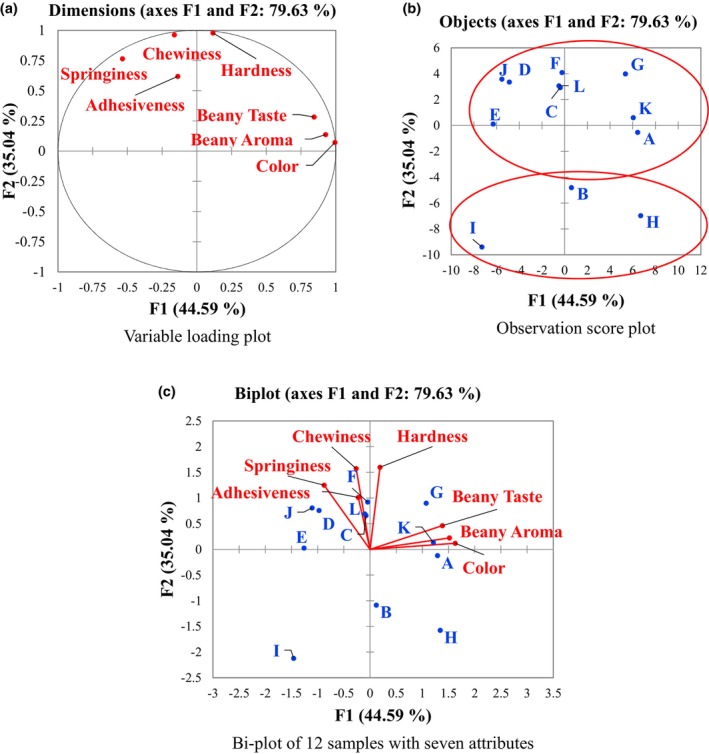
Plots of the first two principal axes of a generalized procrustes analysis (GPA) carried out on the flash profile data. (a, b) Variable loading plot and observation score plot, respectively, (c) biplot of twelve observations with seven variables. F1, first principal component; F2, second principal component. (a) Variable loading plot. (b) Observation score plot. (c) Bi‐plot of 12 samples with seven attributes.

Generalized procrustes analysis of flash profile data was shown in Figure 2. A two‐dimension model was selected and explained 79.63% of total variance with F1, F2 accounting for 44.59%, and 35.04%, respectively.

The variable loading plot (Figure 2a) shows that the selected 12 attributes were separated in the first and second quadrants. The distance between two variables (attributes) in the plot presented their correlations by same direction indicating their positive correlation and otherwise negative correlation. Accordingly, beany aroma, beany taste, and color were all located on the positive site of the F1, indicating that there was a positive correlation between these three attributes. Similarly, chewiness, hardness, springiness, and adhesiveness were located on the positive site of the F2, indicating that there was a positive correlation between these attributes.

The observation score plot (Figure 2b) assessed the similarities and differences between samples by far distance indicating high level of differences otherwise high level of similarities. It shows in Figure 2b, 12 samples were separately distributed in the four quadrants of the plot. Two clusters of them were observed with one cluster containing L, F, J, E, C, D, G, K, A, located on positive side of F2, whereas the other cluster of samples I, B, H on negative side of F2, respectively. As a result, it indicated that samples L, F, J, E, C, D, G, K, and A had similar properties, while the properties of the samples I, B, and H were obviously different from them.

Furthermore, the biplot (Figure 2c) represents the positive correlation between variables (attributes) and observations (samples) with same directions and near distance between them, otherwise there is a negative correlation between them. It shows in the biplot that samples G, K, and A were positively correlated with three attributes including beany aroma, beany taste, and color due to their close distances and same directions. Based on the same principle, Figure 2c shows that samples L, F, C, E, J, and D had a high positive correlation with textural properties including hardness, chewiness, springiness, and adhesiveness. Furthermore, samples H (PNK1), B (PK1), and I (SK1) located far away from aroma, color, and texture attributes, signified that the appearance, aroma, texture, and taste properties of these three samples were not distinct.

Moreover, Figure S2a,b indicated the consensus level of 50 participants on the attributes and samples by overlapping level of the attributes and samples loading points got from each participant, respectively. The GPA map (Figure S2a) indicated the sensory results got from fifty participants reached high consensus level. For instance, the loading points of “Beany Aroma,” “Beany Taste,” and “Color” generated from the participants were often mixed and overlapped indicating that the fifty untrained participants reaching a high level of agreement when they evaluated these attributes of the same products. In addition, the sample loadings in Figure S2b got from different participants of the same product were presented in sample color, and 12 samples were colored differently. It was observed in the plot that, the loadings of same product generated from 50 participants were closely located or highly overlapped. For example, the dots in brown color which represented sample H concentrated on the fourth quadrant, and other samples also followed the same pattern. Therefore, Figure S2 showed that the sensory results obtained by the fifty untrained participants were reliable because of the high repeatability of the data and the high degree of consensus among participants.

#### 3.6.2 Consumer acceptance of compound gels

The results of consumer acceptance test show in Table 4 indicated that the consumers’ acceptance for the 12 samples were generally low. Moreover, PNK2 (5.04), PK2 (5.94), and SK2 (5.36) had the highest acceptance score in each protein group, suggesting that the compound gel could gain higher level of consumer preference when the ratio of protein to KGM was 80:20. Surprisingly, the acceptance scores of PK2 based on all attributes were the highest among all the samples. The acceptance scores of appearance, aroma, texture, overall liking reached 6.18, 6.12, 5.68, and 5.94, respectively.

**TABLE 4:** Results of consumer acceptance test for different compound gels.

Products	PI/KGM ratio (%)	Appearance liking	Aroma liking	Texture liking	Taste liking	Overall liking
PNK1	90:10	3.78 ± 1.80^b^	4.34 ± 1.92^a^	4.24 ± 1.92^b^	4.38 ± 2.08^ab^	4.06 ± 1.87^b^
PNK2	80:20	5.20 ± 1.63^a^	4.82 ± 1.78^a^	5.00 ± 1.62^a^	4.72 ± 1.43^a^	5.04 ± 1.52^a^
PNK3	70:30	4.72 ± 1.57^a^	4.34 ± 1.84^a^	4.34 ± 1.61^ab^	3.70 ± 1.58^b^	4.06 ± 1.57^b^
PNK4	60:40	4.82 ± 1.79^a^	4.38 ± 2.02^a^	4.60 ± 1.63^ab^	4.38 ± 1.65^ab^	4.36 ± 1.61^b^
PK1	90:10	5.30 ± 1.57^b^	5.32 ± 1.65^b^	4.66 ± 1.27^b^	4.60 ± 1.62^b^	4.76 ± 1.52^b^
PK2	80:20	6.18 ± 1.38^a^	6.12 ± 1.84^a^	5.68 ± 1.82^a^	5.50 ± 1.79^a^	5.94 ± 1.67^a^
PK3	70:30	5.38 ± 1.44^b^	5.52 ± 1.49^ab^	5.04 ± 1.18^b^	5.08 ± 1.31^ab^	5.10 ± 1.36^b^
PK4	60:40	5.32 ± 1.27^b^	5.18 ± 1.34^b^	4.82 ± 1.19^b^	4.64 ± 1.48^b^	4.68 ± 1.48^b^
SK1	90:10	4.86 ± 1.98^b^	5.20 ± 1.73^a^	4.32 ± 1.82^b^	4.80 ± 1.74^a^	4.84 ± 1.68^a^
SK2	80:20	5.88 ± 1.65^a^	5.34 ± 1.78^a^	5.54 ± 1.62^a^	5.24 ± 1.68^a^	5.36 ± 1.51^a^
SK3	70:30	5.82 ± 1.49^a^	5.30 ± 1.40^a^	5.20 ± 1.25^a^	5.24 ± 1.32^a^	5.24 ± 1.30^a^
SK4	60:40	5.82 ± 1.57^a^	5.18 ± 1.70^a^	5.42 ± 1.37^a^	4.94 ± 1.52^a^	5.04 ± 1.35^a^

*Note*: 1 = Dislike extremely, 5 = neither like nor dislike, 9 = like extremely. Values expressed are mean ± standard deviation. Data of different alphabets in the same column were different with statistical significance (*p* < .05).

Abbreviations: PI, protein isolate; PNKn (*n* = 1, 2, 3, 4), the protein contained is a peanut protein isolate; PKn (*n* = 1, 2, 3, 4), the protein contained is a pea protein isolate; SKn (*n* = 1, 2, 3, 4), the protein contained is a soy protein isolate.

## 4 DISCUSSION

### 4.1 Effect of KGM on thermal properties of plant protein isolates

Generally, the analysis of thermodynamic properties or transition temperatures of food materials is necessary for food manufacturers because phase transition frequently involved in food processing, storage, or consumption (Parniakov et al., 2018; Reddy et al., 2017). Differential scanning calorimetry of proteins and their mixture slurries with KGM (30% protein or mixture w/w, 5°C/min heating rate) provided insights into the thermal properties of these conjugates (Nawrocka et al., 2017). A higher *T*_d_ value represents that the sample has a higher degree of thermal stability and less structural damage (Guo et al., 2019; Lan et al., 2015). Hence, the results demonstrated that the addition of KGM could significantly reinforce the thermal stability of PNPI (Table 1), which may be due to the large polymer networks formed by PNPI and KGM after the gelation process (Ran et al., 2022). During gelation, hydrophobic interactions, disulfide bond, electrostatic attraction force, and hydrogen bond formation will overcome the repulsive force among peptide molecules, peptide chains, and polysaccharides, thus enhancing the thermal stability of protein through the formation of a complex network between protein and polysaccharide (Zhao et al., 2018). This phenomenon was consistent with the results of studies on bean proteins, that is, samples with greater thermal stability had better gelling performance (Tang, 2008; Yin et al., 2010). However, the maximum values of these thermal denaturation temperatures of PPI and SPI groups were not significantly different from those without KGM, indicating that KGM could not effectively affect the denaturation performance of PPI and SPI. This phenomenon could be explained by the complex changes in the molecular structure of compound gels caused by the interaction between polysaccharide and different proteins. Hence, further studies on the internal binding of each compound gel are needed.

### 4.2 Effect of KGM on the quality of plant protein isolates

Little information is available on using rapid viscosity analysis (RVA) for measuring quality of protein‐based materials, but Ragaee and Abdel‐Aal (2006) found that RVA could be used to characterize proteins in non‐wheat cereal grains. In this experiment, the second peak of the RVA result was used to determine the quality of the protein and KGM mixture based on protein denaturation when it is heated (Ragaee & Abdel‐Aal, 2006). Among RVA results, *PV* indicates the ability of mixed powder to swell freely before the occurrence of physical breakdown; *TV* is an indication of the reduction in viscosity of compounds after *PV* because of the strong decomposition of protein or/and polysaccharide granules by high temperature; and *FV* is the final viscosity of the compound gels (Oladele & Aina, 2007). Through the results, the addition of KGM to PI could generally enhanced the *PV*, *TV*, and *FV* values, which was contrary to the results of a previous paper on modifying flaxseed gum (polysaccharide) with KGM (polysaccharide) which demonstrated that the addition of KGM to flaxseed gum generally reduced *PV*, *TV*, and *FV* values (Jiang et al., 2019). One possible explanation is that the electrostatic interactions between proteins and polysaccharide in water can lead to the formation of hydrogen bonds and covalent bonds, contributing to the increased viscosity (Jiang et al., 2019). Furthermore, the trend and the value of RVA parameters varied among PI groups, indicating significant variations in protein properties when subjected to heating and cooling treatments because of the interactions of PI and KGM during agitation were complex and unpredictable (Ragaee & Abdel‐Aal, 2006; Wang et al., 2018).

All in all, an appropriate amount of KGM could be added to soy/peanut/pea protein isolates because it could significantly improve the thickening ability of its compound gels by affecting the viscosity of these three types of plant proteins. This finding was consistent with the previous results stated by Ding et al. (2003), thus allowing these proteins to be used as stabilizers or thickeners in healthier foods, such as low‐fat or reduced‐fat meat products (Lin & Mei, 2000), vegan foods, and plant‐based protein beverages (Valentine et al., 2008; Wei et al., 2020).

### 4.3 Effect of KGM on visual appearance of compound gels

The appearance of different gels (Figure S1) provided a rough concept of the gel texture, which described in Table S1. The results showed that the physical modification performed by mixing KGM with protein showed a tendency to improve the functional characteristics of PNPI, PPI, and SPI. Compared to the previous research on the thickening properties of KGM, the results were quite similar (Yang et al., 2020). Besides, the formation of heterogeneous compound gels may be ascribed to the fact that the content of KGM in PNK5, PK5, and SK5 was too high, and there were obvious geometrical differences between the spherical fat and rod‐shaped polysaccharide molecules, causing the increase in the thermodynamic instability of the system, then phase separation occurred (Dickinson, 2003).

### 4.4 Effect of KGM on textural properties of compound gels

The TPA is a simple and rapid analytical technique which is widely applied to measure how food performs during processing or handling or feels when consumer eat it (Rahman & Al‐Farsi, 2005). Moreover, texture analysis is a critical test in food processing since the texture of a product will determine its characteristics such as mouthfeel, appearance, and stability. For this reason, the texture evaluation is often a necessary step in developing a new food product, optimizing processing variables, and exploring the possible application of food ingredients in food products (Thrimawithana et al., 2010).

In current study, the results stated that the SK4 gel displayed wonderful hardness, chewability, cohesiveness, and springiness. PNK4 gel had considerable hardness, chewiness, and cohesiveness, while springiness was worse than PNK2; PK4 gel which presented great hardness, chewiness, and springiness, while only cohesiveness was not better than PK1. As a result, SK4, PNK4, and PK4 all revealed a great potential to be used in elastic foods such as jelly and sausage, because most of parameters were favorable (Feng & Wu, 2009; Jiang et al., 2019).

### 4.5 Effect of KGM on microstructure features of compound gels

SEM is considered as a convenient technique to observe the internal structure of compound gels directly. As observed, the gel network of PNK1 (Figure 1a), PK1 (Figure 1e), and SK1 (Figure 1i) were relatively loose and fractured, which properly explains the lower values of gel hardness. This may be because the protein undergoes a denaturation process during heat‐induced gelation, where hydrogen bonds and hydrophobic interactions are broken and the protein molecules are unfolded (Ran et al., 2022). Meanwhile, when the PI/KGM ratio was 90:10, the hydrogen bonds and interactions between proteins and polysaccharides were too weak. With increasing KGM proportion in PPI and SPI groups, the pores decreased in number, turning fractured gel network into cross‐linking state (Yang et al., 2020). This phenomenon might be because KGM could interact with PPI or SPI mainly via hydrogen bonds and thereby fill the pores of the PPI or SPI network, making the structure more compact and smoother (Jiang et al., 2019; Ran et al., 2022). The similar results have been demonstrated in the outcome of KGM on modifying soya, wheat, corn protein isolates (Simon et al., 2011). Additionally, SK3 (Figure 1k) and SK4 (Figure 1l) displayed the smoothest texture among twelve samples, specifying that the microstructure of SPI/KGM compound gel was more compact and intense due to the hydrogen bonding efficiently formed between KGM and soy protein molecules (Ding et al., 2003). Nevertheless, among PNPI/KGM group, if the proportion of KGM continued to increase, the gel network became more fragmented (Figure 1c,d). This adverse effect may be due to the saturation of the cross‐linking zones and the aggregation of polymer groups, which would destroy the cohesive structure of the systems (Thrimawithana et al., 2010).

### 4.6 Effect of KGM on sensory properties of compound gels

The flash profile, a modern sensory profiling technique, is a combination of collecting of attributes and sorting, based on the simultaneous introduction of all the samples to be assessed, which allows a rapid sensory profile with high discriminatory capability become possible, obtaining a considerable amount and variety of attributes (Delarue & Sieffermann, 2004; Marques et al., 2019). As shown in Figure 2b, samples H (PNK1), B (PK1), and I (SK1) were significantly difference from samples L (PK2), F (PK3), C (PK4), E (SK2), J (SK3), D (SK4), G (PNK4), K (PNK2), and A (PNK3) because the proportion of KGM was insufficient, which indicated that the optimal amount of KGM could significantly change the overall sensory properties of the gel. Samples L, F, C, G, K, and A had similar overall sensory properties as samples E, J, and D, suggesting that PPI and PNPI could achieve similar sensory properties with SPI/KGM after modification (Gkatzionis et al., 2013). According to Figure 2c, samples L, F, C, E, J, and D had a high positive correlation with textural properties which confirmed that adding KGM to PPI could significantly improve the textural attributes of the final product, which were consistent with the TPA results (Wangcharoen et al., 2016). Samples G, K, and A were highly positively correlated with beany flavor and taste, which may be speculated due to the residue of oil in the raw materials. Residues of oil in peanut protein isolate lead to the higher concentrations of odors such as benzaldehyde in peanut group than others (Lee et al., 2020), and the synergistic effect between oil and volatile compounds can intensify the beany odor of the final products as well (Zhang & Chen, 2004). Samples H, B, and I presented a low correlation with the beany aroma and taste, revealing that the odor flavor was not fully derived from the legumes, KGM contributed a large portion of the odor flavor (Li et al., 2007).

A product acceptance test can determine which types of protein and which formula was preferred by consumers when compared to other samples. In this consumer acceptance test, the overall liking scores above 5 were only PNK2 (5.04), PK2 (5.94), PK3 (5.10), SK2 (5.36), SK3 (5.24), and SK4 (5.04) gels, which proved that all samples other than these six varieties were disliked by participants. This result also explained why soy protein isolate was more widely used in the food industry than the other two proteins (Gao et al., 2015). Accordingly, this proved that PK2 and PNK2 had great potential as prototypes of novel plant‐based products and were worthy of further development by scholars. Furthermore, most samples with lower overall acceptance scores had lower aroma and taste acceptance scores, such as PNK3 and PNK4, indicating that beany odor and taste could significantly diminish the acceptability of plant‐based products among consumers. Hence, even though the incorporation of KGM can enhance the textural properties of protein‐based products, it remains imperative to address the presence of beany odor before adding bean proteins as food ingredients (Dai et al., 2007).

## 5 CONCLUSIONS

This study provides clear information about the physiochemical features and sensory properties of three types of mixed gels with different ratios of protein isolate/polysaccharide. The results indicated that PNPI/KGM showed significant differences in thermal properties and protein quality with PI samples, while the synergistic effect between SPI or PPI and KGM was not obvious in the modification of thermal properties for compound gels, but still improved to some extent. The additional of an optimal amount of KGM significantly changed the hardness and chewiness characters of three compound gels, which matched the sensory results. Besides, PK2 and PNK2 had great potential to be viewed as prototypes of novel plant‐based products because of the highest consumer acceptance scores.

All these results revealed that KGM modification in a certain proportion could significantly improve both physiochemical and sensory characteristics of protein‐based foods, so that broadening the application range of PNPI, PPI, and SPI. However, the nutritional characteristics and the digestibility of the compound gels need to be further studied.

## AUTHOR CONTRIBUTIONS

**Yueying Yao:** Data curation (equal); formal analysis (equal); investigation (equal); validation (equal); writing – original draft (equal). **Wenmeng He:** Formal analysis (equal); methodology (equal); software (equal); validation (equal); visualization (equal); writing – original draft (equal). **Baojun Xu:** Conceptualization (equal); funding acquisition (equal); methodology (equal); project administration (equal); resources (equal); supervision (equal); visualization (equal); writing – review and editing (equal).

## CONFLICT OF INTEREST STATEMENT

All authors claim that there is no conflict of interest in this manuscript.

## Supporting information


Table S1.
Figure S1.
Figure S2.

## Data Availability

The data that support the findings of this study are available on request from the corresponding author.
