# Supplementary material for: Physiochemical characteristics and sensory properties of plant protein isolates–konjac glucomannan compound gels
Source: Food Sci Nutr. 2023 Jun 9;11(9):5063–77. doi: 10.1002/fsn3.3471 (PMC10494608; doi:10.1002/fsn3.3471)
Supplement: Supplementary file 1 — Table S1. Figure S1. Figure S2. [file FSN3-11-5063-s001.docx]

Supplemental **Table S1.** Appearance description of different types of compound gels

| Samples | PI/KGM ratio (%) | Appearance |
| --- | --- | --- |
| PNPI | 100:0 | Liquid with highest turbidity |
| PNK1 | 90:10 | Softness, easy to flow, poor formability |
| PNK2 | 80:20 | Slightly firm, relatively smooth surface |
| PNK3 | 70:30 | Firmness, smooth surface |
| PNK4 | 60:40 | Firmness, smooth surface |
| PNK5 | 50:50 | Firmness outside, but the inside is still an uneven solid-liquid mixture |
| PPI | 100:0 | Liquid with relatively high turbidity |
| PK1 | 90:10 | Softness, easy to flow |
| PK2 | 80:20 | Slightly firm, relatively smooth surface |
| PK3 | 70:30 | Firmness, smooth surface |
| PK4 | 60:40 | Firmness, smooth surface |
| PK5 | 50:50 | Firmness outside, but the inside is still an uneven solid-liquid mixture |
| SPI | 100:0 | Little transparent liquid |
| SK1 | 90:10 | Softness, easy to flow, poor formability |
| SK2 | 80:20 | Firmness, smooth surface |
| SK3 | 70:30 | Firmness, smooth surface |
| SK4 | 60:40 | Firmness, smooth surface |
| SK5 | 50:50 | Firmness outside, a part of inside was an uneven solid-liquid mixture |

^*^ PI, protein isolate; PNPI, peanut protein isolate; PPI, pea protein isolate; SPI, soy protein isolate; PNKn (n = 1, 2, 3, 4, 5) means the protein contained is peanut protein isolate; PKn (n = 1, 2, 3, 4, 5) means the protein contained is pea protein isolate; SKn (n = 1, 2, 3, 4, 5) means the protein contained is soy protein isolate.


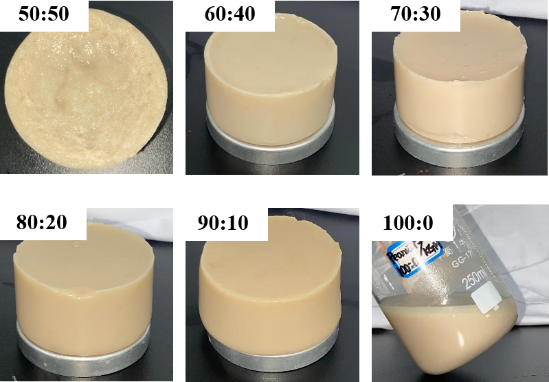

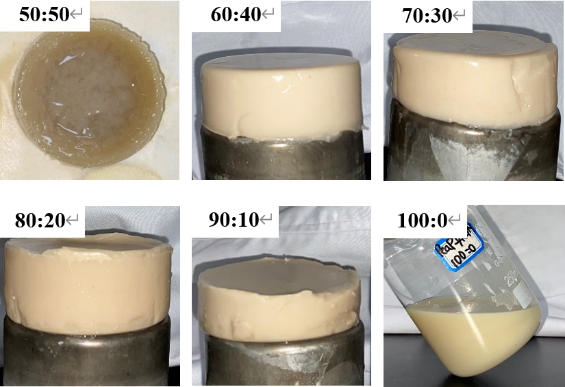


A) Appearance of the PNPI/KGM gels B) Appearance of the PPI/KGM gels
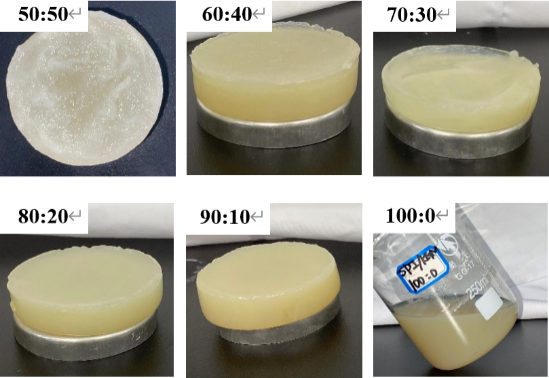


C) Appearance of the SPI/KGM gels

Supplemental **Fig. S1.** Appearance of the PNPI/KGM, PPI/KGM, and SPI/KGM gels.

a) Variables plot reflects the consensus of 50 participants on the attributes of 12 products

b) Objects plot reflects the consensus of 50 participants for 12 products

Supplemental **Fig. S2.** Plots of the first two principal axes of a generalized procrustes analysis carried out on the Flash Profile data. a) variables plot reflects the consensus of 50 participants on the attributes of 12 products, b) observations plot reflects the consensus of 50 participants for 12 products. F1: first principal component, F2: second principal component.
